# Supplementary material for: Mesenchymal Stem Cells Delivered Locally to Ischemia-Reperfused Kidneys via Injectable Hyaluronic Acid Hydrogels Decrease Extracellular Matrix Remodeling 1 Month after Injury in Male Mice
Source: Cells. 2023 Jul 4;12(13):1771. doi: 10.3390/cells12131771 (PMC10340256; doi:10.3390/cells12131771)
Supplement: Supplementary file 1 [file cells-12-01771-s001.zip › cells-2293162-supplementary.pdf]

Supplementary Materials:

Supplemental Figure S1:

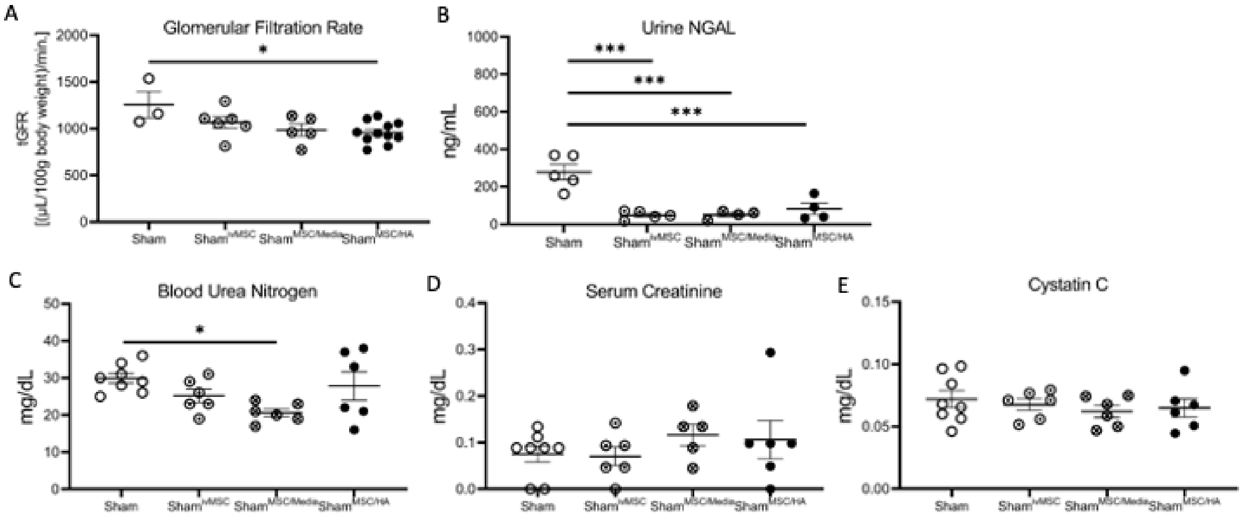

Supplemental Figure S1 Legend:

Renal functional outcomes at sacrifice, 1 month after sham bilateral ischemia-reperfusion acute kidney injury. Groups received treatment of either MSCs via iv (Sham^{MSCiv}), MSCs injected under the left kidney capsule (Sham$^{MSC/media}$) or MSCs encapsulated in HA hydrogel delivered under the left kidney capsule (Sham$^{MSC/HA}$) 3 days after AKI. Measured tGFR (A), Urine NGAL (B), blood urea nitrogen (C), serum Creatinine (D) and serum cystatin C (E). * indicates $p < 0.05$, ** indicates $p < 0.01$; *** indicates $p < 0.001$.

Supplemental Figure S2:

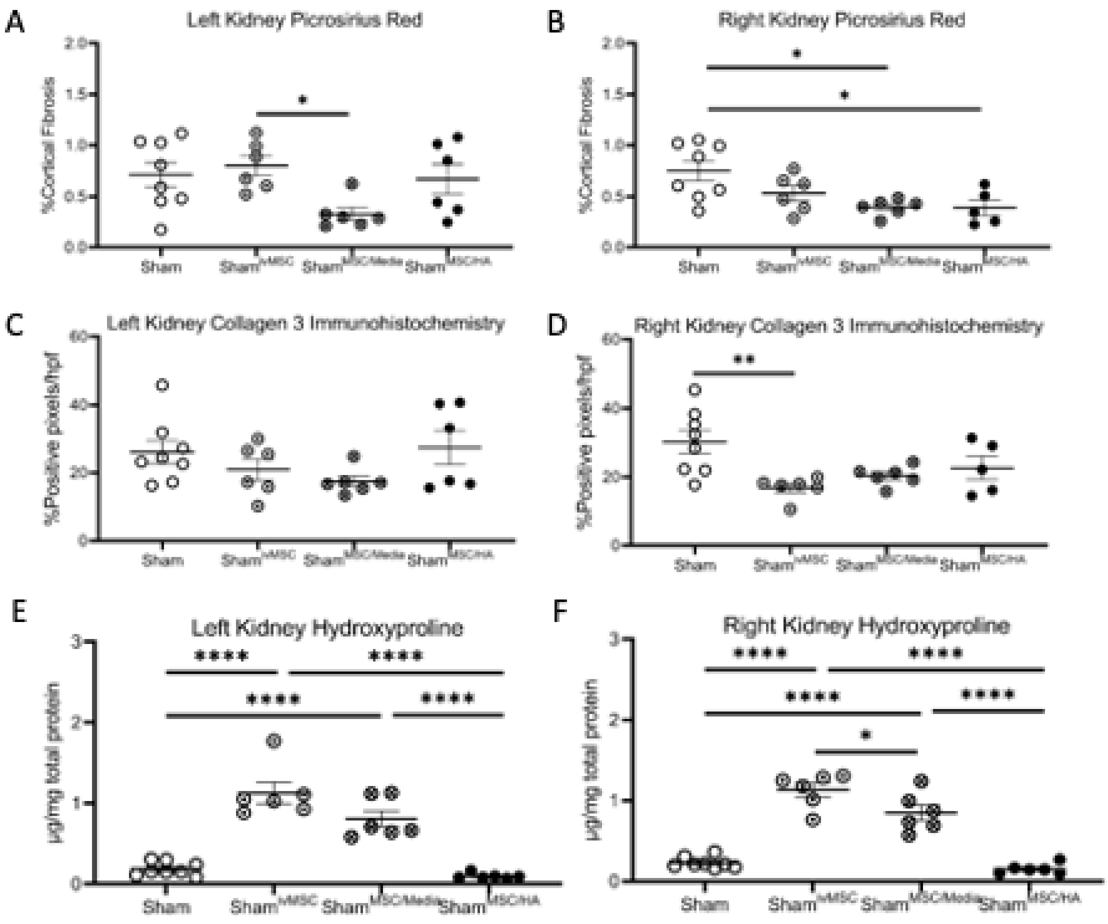

Supplemental Figure S2 Legend: : Kidney fibrosis outcomes at sacrifice, 1 month after sham bilateral ischemia-reperfusion acute kidney injury in the various treatment groups. Quantification of cortical fibrosis via polarized light after Picrosirius Red staining in the left (A) and right (B) kidneys. Quantification of immunohistochemistry staining for Collagen 3 in the left (C) and right (D) kidneys. Hydroxyproline content of the left (E) and right (F) kidneys. The sham treatment groups demonstrate that MSCs devivered via HA did not result in kidney fibrosis. * indicates $p < 0.05$, ** indicates $p < 0.01$, **** indicates $p < 0.0001$.

Supplemental Figure S3:

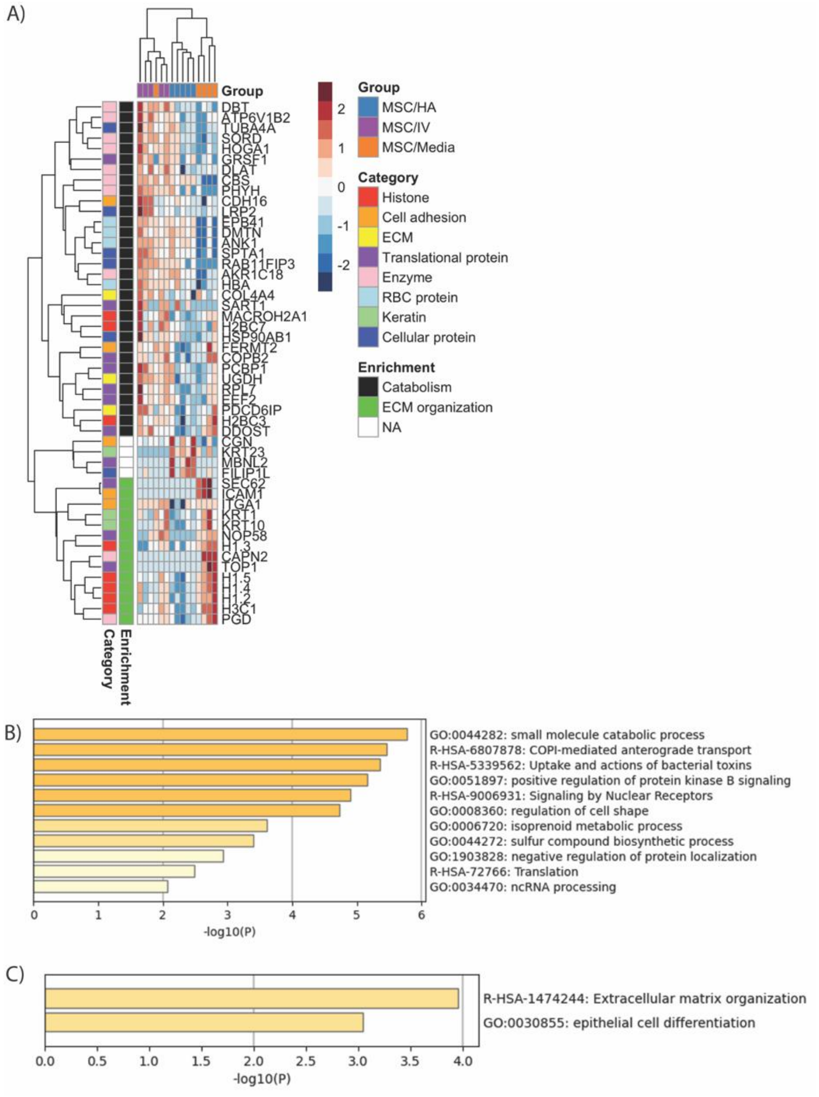

Supplemental Figure S3 Legend: A). Heatmap showing grouping of treated kidneys (X-axis) according to their relative levels of significant proteins also colored by general protein category (Y-axis). Also colored by enrichment of proteins by treatment group from B) and C). B) Enrichment results of proteins expressed at significantly elevated levels in MSC/IV treated kidneys showing term (Y-axis) and -log10(pvalue) (X-axis) based on number of proteins enriched. C) Enrichment terms of proteins expressed elevated levels in MSC/Media treated kidneys.

Supplemental Figure S4:

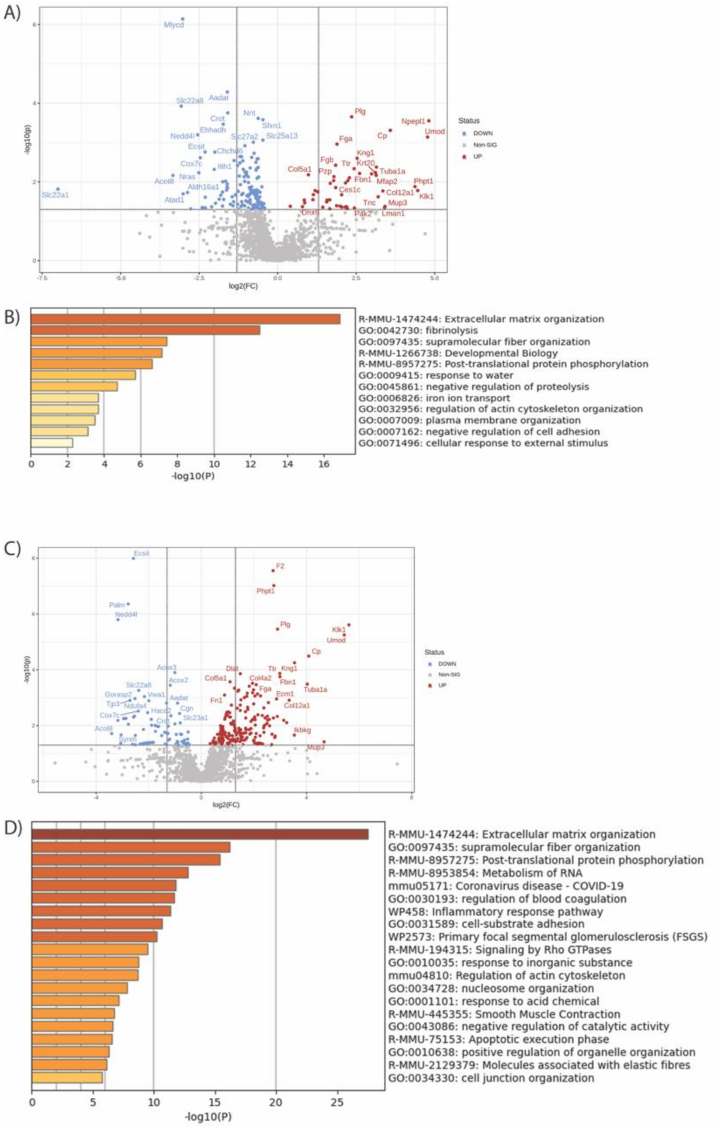

Supplemental Figure S4 Legend: A) Volcano plot comparing Sham vs. MSC/HA treated kidneys, with B) enrichment terms of proteins expressed at significantly elevated levels in MSC/HA treated kidneys. C) Volcano plot comparing Sham vs. MSC/IV treated kidneys, with B) enrichment terms of proteins expressed at significantly elevated levels in MSC/IV treated kidneys.

Supplemental Figure S5:

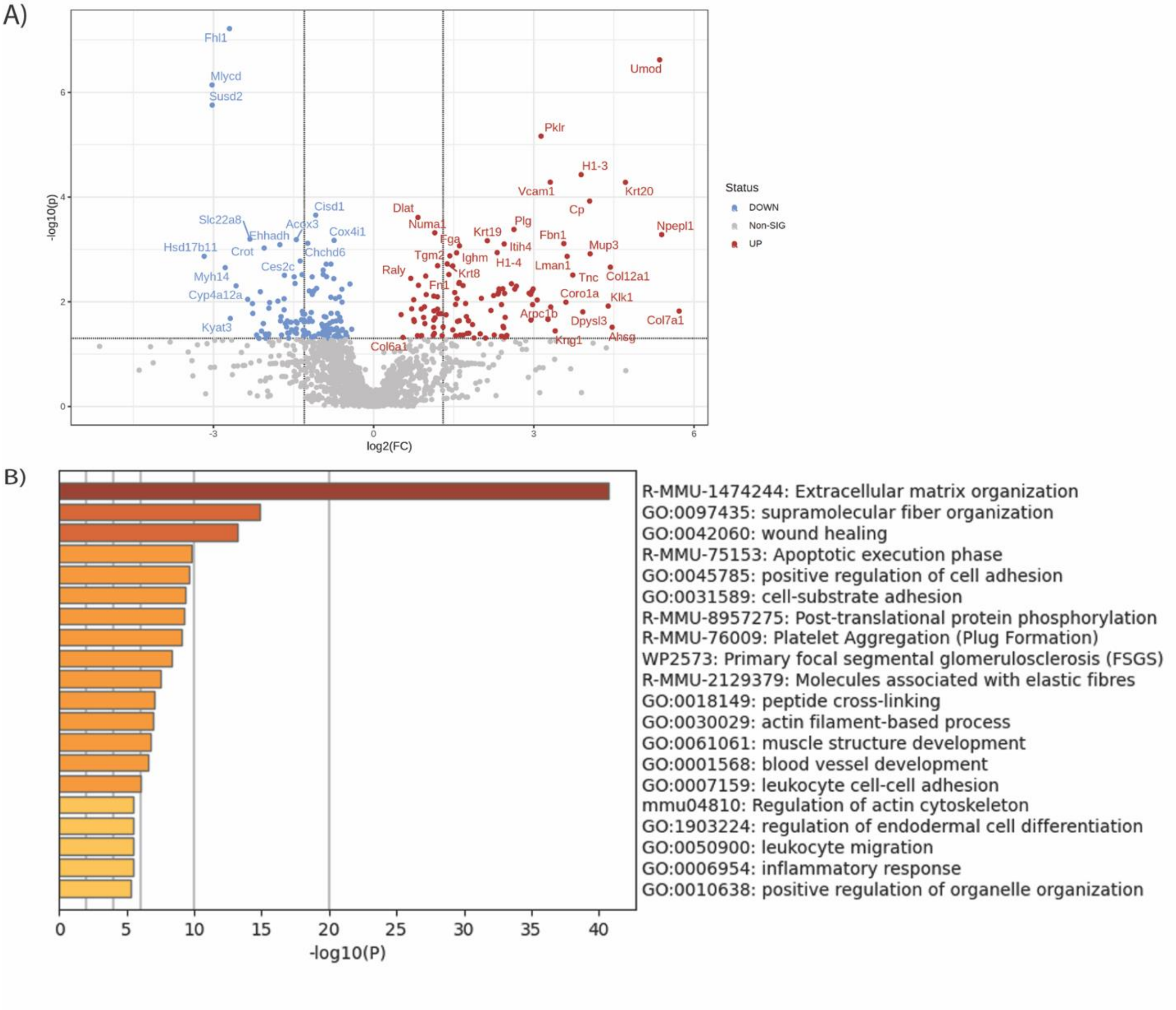

Supplemental Figure S5 Legend: A) Volcano plot comparing Sham vs. MSC/media treated kidneys, with B) enrichment terms of proteins expressed at significantly elevated levels in MSC/media treated kidneys.

Supplemental Figure S6:

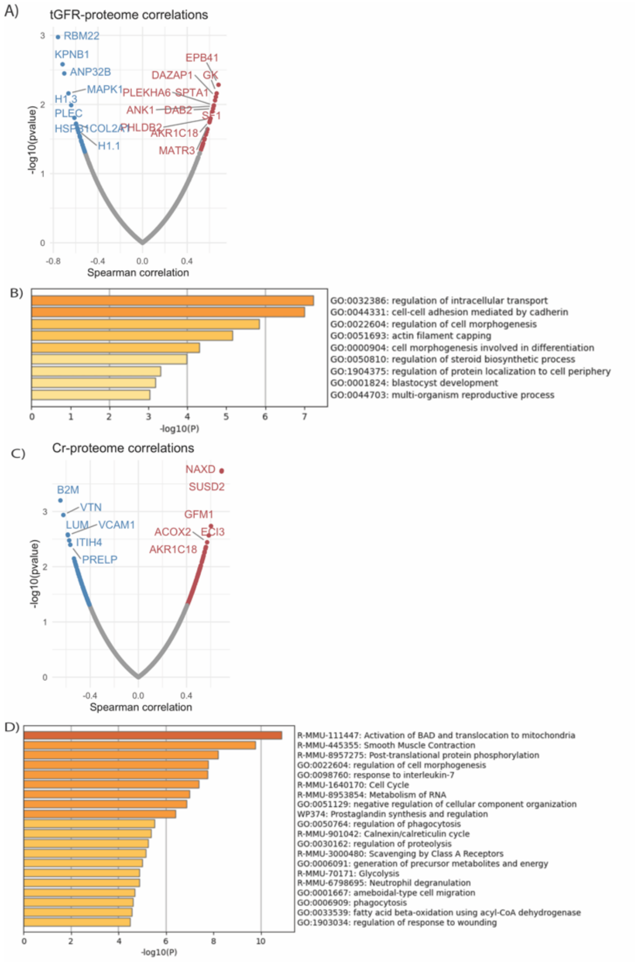

Supplemental Figure S6: A) Correlation U-plot of tGFR vs. protein expression levels with spearman correlation value on X-axis and -log10(pvalue) on Y-axis showing positively (red) and negatively (blue) correlated proteins with tGFR. B) Enrichment results of significantly and highly correlated proteins with tGFR, showing enrichment terms and -log10(pvalue). C) Correlation U-plot of Creatinine vs. protein expression levels with spearman correlation value on X-axis and -log10(pvalue) on Y-axis showing positively (red) and negatively (blue) correlated proteins with Creatinine. D) Enrichment results of significantly and highly correlated proteins with Creatinine, showing enrichment terms and -log10(pvalue).

Supplemental Figure S7:

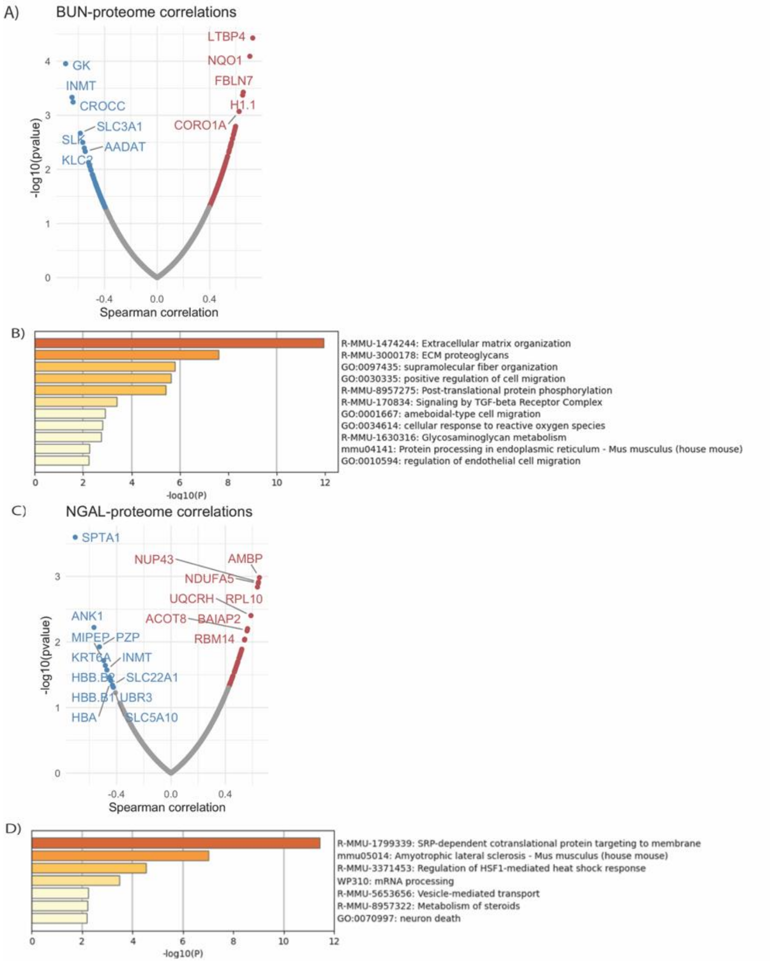

Supplemental Figure S7: A) Correlation U-plot of BUN vs. protein expression levels with spearman correlation value on X-axis and -log10(pvalue) on Y-axis showing positively (red) and negatively (blue) correlated proteins with BUN. B) Enrichment results of significantly and highly correlated proteins with BUN, showing enrichment terms and -log10(pvalue). C) Correlation U-plot of NGAL vs. protein expression levels with spearman correlation value on X-axis and -log10(pvalue) on Y-axis showing positively (red) and negatively (blue) correlated proteins with NGAL. D) Enrichment results of significantly and highly correlated proteins with NGAL, showing enrichment terms and -log10(pvalue).

Supplemental Table S1: Top 100 significantly different proteins among all groups

Protein	obs.tot	sumsq.between	sumsq.within	statistic	pvalue	fdr
UMOD	25	20.14	3.86	26.12	1.08E-07	1.91E-04
CP	25	19.87	4.13	24.04	2.13E-07	1.91E-04
MLYCD	25	17.75	6.25	14.19	1.21E-05	7.23E-03
TTR	25	17.20	6.80	12.65	2.72E-05	1.22E-02
FBN1	25	16.64	7.36	11.31	5.81E-05	2.09E-02
DLAT	25	16.32	7.68	10.64	8.73E-05	2.61E-02
AMBP	25	16.06	7.94	10.12	1.20E-04	2.75E-02
KRT19	25	15.88	8.12	9.78	1.49E-04	2.75E-02
COL5A1	25	15.88	8.12	9.78	1.50E-04	2.75E-02
H1.4	25	15.83	8.17	9.70	1.58E-04	2.75E-02
APOA4	25	15.78	8.22	9.60	1.68E-04	2.75E-02
COL12A1	25	15.67	8.33	9.40	1.92E-04	2.88E-02
PKLR	25	15.41	8.59	8.96	2.57E-04	3.54E-02
PLG	25	15.31	8.69	8.80	2.87E-04	3.54E-02
ACOT8	25	15.26	8.74	8.73	3.02E-04	3.54E-02
ALB	25	15.22	8.78	8.67	3.15E-04	3.54E-02
CYP4A12A	25	15.13	8.87	8.52	3.48E-04	3.57E-02
NEDD4L	25	15.05	8.95	8.41	3.77E-04	3.57E-02
TNC	25	15.05	8.95	8.41	3.78E-04	3.57E-02
SLC22A8	25	14.89	9.11	8.17	4.50E-04	4.04E-02
TUBA1A	25	14.61	9.39	7.77	5.98E-04	5.11E-02
COL3A1	25	14.44	9.56	7.56	7.02E-04	5.74E-02
PDCD6IP	25	14.35	9.65	7.43	7.71E-04	6.00E-02
TOR1AIP1	25	14.31	9.69	7.38	8.02E-04	6.00E-02
NUMA1	25	14.23	9.77	7.29	8.62E-04	6.08E-02
COL1A1	25	14.18	9.82	7.22	9.10E-04	6.08E-02
COX7C	25	14.17	9.83	7.21	9.14E-04	6.08E-02
PZP	25	14.13	9.87	7.16	9.53E-04	6.12E-02
COL1A2	25	14.09	9.91	7.11	9.87E-04	6.12E-02
FGA	25	14.05	9.95	7.06	1.03E-03	6.18E-02
COL5A2	25	13.90	10.10	6.88	1.19E-03	6.60E-02
ATAD1	25	13.87	10.13	6.84	1.22E-03	6.60E-02
CROT	25	13.84	10.16	6.81	1.25E-03	6.60E-02
FGB	25	13.79	10.21	6.75	1.31E-03	6.60E-02
B2M	25	13.78	10.22	6.74	1.33E-03	6.60E-02
FN1	25	13.76	10.24	6.72	1.34E-03	6.60E-02

EHHADH	25	13.74	10.26	6.69	1.37E-03	6.60E-02
H1.5	25	13.71	10.29	6.67	1.40E-03	6.60E-02
H1.3	25	13.68	10.32	6.63	1.45E-03	6.60E-02
ICAM1	25	13.66	10.34	6.61	1.47E-03	6.60E-02
ECSIT	25	13.61	10.39	6.55	1.54E-03	6.74E-02
SPTA1	25	13.51	10.49	6.43	1.69E-03	7.25E-02
MATN2	25	13.43	10.57	6.35	1.81E-03	7.56E-02
ITIH4	25	13.36	10.64	6.27	1.93E-03	7.89E-02
H1.2	25	13.31	10.69	6.22	2.01E-03	8.04E-02
FGG	25	13.25	10.75	6.16	2.12E-03	8.17E-02
KRT8	25	13.24	10.76	6.15	2.14E-03	8.17E-02
CES1C	25	13.19	10.81	6.11	2.22E-03	8.23E-02
CSRP1	25	13.18	10.82	6.09	2.25E-03	8.23E-02
TGM2	25	13.16	10.84	6.07	2.29E-03	8.23E-02
MFAP2	25	13.13	10.87	6.04	2.34E-03	8.26E-02
VWA1	25	13.00	11.00	5.91	2.62E-03	9.05E-02
NPEPL1	25	12.96	11.04	5.87	2.71E-03	9.18E-02
RUVBL2	25	12.93	11.07	5.84	2.78E-03	9.25E-02
MUG1	25	12.89	11.11	5.80	2.89E-03	9.32E-02
EPB41	25	12.88	11.12	5.79	2.90E-03	9.32E-02
GNS	25	12.74	11.26	5.66	3.25E-03	1.02E-01
CHCHD6	25	12.73	11.27	5.65	3.29E-03	1.02E-01
KLK1	25	12.71	11.29	5.63	3.34E-03	1.02E-01
CGN	25	12.67	11.33	5.59	3.44E-03	1.02E-01
MUP3	25	12.66	11.34	5.59	3.47E-03	1.02E-01
PLEC	25	12.62	11.38	5.55	3.59E-03	1.04E-01
LAMA2	25	12.56	11.44	5.49	3.77E-03	1.08E-01
MEP1B	25	12.51	11.49	5.45	3.92E-03	1.09E-01
KANK3	25	12.51	11.49	5.44	3.93E-03	1.09E-01
MYH10	25	12.49	11.51	5.42	4.00E-03	1.09E-01
KRT23	25	12.47	11.53	5.41	4.06E-03	1.09E-01
COL7A1	25	12.44	11.56	5.38	4.16E-03	1.09E-01
PHPT1	25	12.43	11.57	5.37	4.18E-03	1.09E-01
EMILIN1	25	12.38	11.62	5.33	4.36E-03	1.12E-01
VIM	25	12.33	11.67	5.28	4.53E-03	1.15E-01
CES2C	25	12.27	11.73	5.23	4.75E-03	1.19E-01
FLNA	25	12.24	11.76	5.20	4.88E-03	1.20E-01
PM20D1	25	12.21	11.79	5.18	4.96E-03	1.21E-01
HPX	25	12.19	11.81	5.16	5.06E-03	1.21E-01

DHX9	25	12.16	11.84	5.14	5.16E-03	1.22E-01
ACTN1	25	12.10	11.90	5.08	5.43E-03	1.27E-01
HNRNPAB	25	12.05	11.95	5.04	5.63E-03	1.29E-01
PECR	25	12.05	11.95	5.04	5.65E-03	1.29E-01
ANK1	25	12.03	11.97	5.02	5.74E-03	1.29E-01
AMACR	25	11.99	12.01	4.99	5.90E-03	1.31E-01
ACOX2	25	11.95	12.05	4.96	6.07E-03	1.31E-01
ECI3	25	11.94	12.06	4.95	6.11E-03	1.31E-01
H3C1	25	11.93	12.07	4.94	6.20E-03	1.31E-01
KNG1	25	11.92	12.08	4.94	6.21E-03	1.31E-01
PGD	25	11.87	12.13	4.89	6.47E-03	1.35E-01
LAMA3	25	11.84	12.16	4.87	6.60E-03	1.35E-01
ANXA2	25	11.84	12.16	4.87	6.63E-03	1.35E-01
LUC7L2	25	11.78	12.22	4.82	6.90E-03	1.39E-01
CAPG	25	11.77	12.23	4.82	6.95E-03	1.39E-01
KLC4	25	11.75	12.25	4.79	7.10E-03	1.39E-01
ACY1	25	11.74	12.26	4.79	7.12E-03	1.39E-01
PCBP1	25	11.71	12.29	4.77	7.27E-03	1.41E-01
H1.1	25	11.69	12.31	4.75	7.41E-03	1.42E-01
KRT20	25	11.63	12.37	4.70	7.71E-03	1.46E-01
PDLIM7	25	11.58	12.42	4.67	8.00E-03	1.49E-01
UACA	25	11.58	12.42	4.66	8.02E-03	1.49E-01
ITGB1	25	11.56	12.44	4.65	8.14E-03	1.49E-01
MTA2	25	11.54	12.46	4.63	8.28E-03	1.49E-01

Supplemental Table S2: Top 100 significantly different proteins among treatment groups

Protein	obs.tot	sumsq.between	sumsq.within	statistic	pvalue	fdr
MBNL2	15	10.04	5.03	11.97	1.39E-03	2.26E-01
KRT23	15	9.21	4.76	11.62	1.56E-03	2.34E-01
GRSF1	15	10.27	6.83	9.02	4.06E-03	5.54E-01
RPL7	15	7.91	5.37	8.84	4.37E-03	5.54E-01
H1.4	15	4.83	3.33	8.70	4.62E-03	5.54E-01
NOP58	15	9.04	6.41	8.46	5.10E-03	5.73E-01
ANK1	15	7.15	5.38	7.97	6.27E-03	6.22E-01
FILIP1L	15	6.63	5.04	7.90	6.47E-03	6.22E-01
AKR1C18	15	7.81	5.96	7.86	6.58E-03	6.22E-01
H1.2	15	4.69	3.64	7.73	6.98E-03	6.22E-01
EPB41	15	12.05	9.47	7.63	7.26E-03	6.22E-01
ITGA1	15	4.68	3.90	7.19	8.84E-03	6.35E-01
MACROH2A1	15	5.74	5.02	6.86	1.03E-02	6.35E-01
KRT1	15	6.74	5.99	6.76	1.08E-02	6.35E-01
RAB11FIP3	15	8.30	7.45	6.68	1.12E-02	6.35E-01
PCBP1	15	7.11	6.42	6.65	1.14E-02	6.35E-01
H1.5	15	3.92	3.55	6.63	1.15E-02	6.35E-01
CBS	15	7.54	6.86	6.59	1.17E-02	6.35E-01
H3C1	15	7.98	7.63	6.28	1.36E-02	6.35E-01
FERMT2	15	2.44	2.34	6.24	1.39E-02	6.35E-01
H2BC3	15	6.19	6.16	6.03	1.54E-02	6.35E-01
CAPN2	15	8.00	8.00	6.00	1.56E-02	6.35E-01
SPTA1	15	2.34	2.41	5.84	1.70E-02	6.35E-01
SEC62	15	5.79	5.97	5.83	1.71E-02	6.35E-01
DBT	15	9.49	9.90	5.75	1.77E-02	6.35E-01
PGD	15	8.39	8.82	5.71	1.81E-02	6.35E-01
ICAM1	15	5.32	5.59	5.71	1.81E-02	6.35E-01
SART1	15	7.73	8.25	5.63	1.89E-02	6.35E-01
H2BC7	15	4.58	4.90	5.60	1.91E-02	6.35E-01
HBA	15	3.71	4.02	5.54	1.98E-02	6.35E-01
DMTN	15	6.86	7.48	5.50	2.01E-02	6.35E-01
CDH16	15	8.11	8.96	5.43	2.09E-02	6.35E-01
KRT10	15	6.42	7.10	5.43	2.10E-02	6.35E-01
DLAT	15	5.35	5.92	5.42	2.10E-02	6.35E-01
SORD	15	8.26	9.25	5.36	2.17E-02	6.35E-01
HSP90AB1	15	8.32	9.56	5.22	2.34E-02	6.35E-01

COL4A4	15	7.19	8.51	5.07	2.53E-02	6.35E-01
HOGA1	15	7.42	8.85	5.03	2.59E-02	6.35E-01
LRP2	15	8.80	10.57	5.00	2.64E-02	6.35E-01
COPB2	15	6.47	7.79	4.99	2.66E-02	6.35E-01
DDOST	15	6.26	7.58	4.96	2.69E-02	6.35E-01
ATP6V1B2	15	9.08	10.99	4.95	2.70E-02	6.35E-01
H1.3	15	5.16	6.29	4.92	2.75E-02	6.35E-01
TOP1	15	6.98	8.52	4.91	2.76E-02	6.35E-01
CGN	15	4.79	5.89	4.88	2.82E-02	6.35E-01
PDCD6IP	15	5.41	6.67	4.87	2.83E-02	6.35E-01
EEF2	15	8.37	10.35	4.86	2.85E-02	6.35E-01
PHYH	15	7.14	8.97	4.78	2.98E-02	6.35E-01
TUBA4A	15	8.75	11.01	4.77	2.99E-02	6.35E-01
UGDH	15	4.90	6.17	4.77	3.00E-02	6.35E-01
RDX	15	5.59	7.06	4.75	3.02E-02	6.35E-01
AP2M1	15	6.88	8.70	4.74	3.03E-02	6.35E-01
DDX39B	15	3.66	4.64	4.73	3.06E-02	6.35E-01
CCT7	15	7.90	10.07	4.71	3.09E-02	6.35E-01
RPL19	15	4.55	5.80	4.71	3.09E-02	6.35E-01
VIL1	15	9.15	11.69	4.70	3.11E-02	6.35E-01
MLEC	15	5.48	7.00	4.70	3.12E-02	6.35E-01
RPS3A	15	5.07	6.50	4.68	3.15E-02	6.35E-01
RHOA	15	6.40	8.31	4.62	3.25E-02	6.35E-01
ASS1	15	8.38	10.92	4.60	3.28E-02	6.35E-01
ANK3	15	7.94	10.38	4.59	3.30E-02	6.35E-01
CTTN	15	6.76	8.91	4.55	3.38E-02	6.35E-01
TUFM	15	7.51	9.91	4.55	3.39E-02	6.35E-01
RPS6	15	5.07	6.70	4.54	3.41E-02	6.35E-01
KANK3	15	8.68	11.49	4.53	3.42E-02	6.35E-01
IDH2	15	8.13	10.87	4.49	3.50E-02	6.35E-01
IDH3A	15	5.10	6.86	4.46	3.56E-02	6.35E-01
ENO1	15	6.06	8.17	4.45	3.58E-02	6.35E-01
RPL8	15	5.27	7.19	4.40	3.68E-02	6.35E-01
PALM	15	4.99	6.84	4.37	3.75E-02	6.35E-01
CPT1A	15	9.10	12.53	4.36	3.78E-02	6.35E-01
COL4A3	15	5.99	8.27	4.35	3.80E-02	6.35E-01
MRPS21	15	6.09	8.42	4.34	3.82E-02	6.35E-01
PC	15	8.90	12.37	4.32	3.87E-02	6.35E-01
EIF2S3X	15	4.01	5.58	4.31	3.89E-02	6.35E-01

FHL1	15	7.43	10.41	4.28	3.95E-02	6.35E-01
RPL4	15	4.68	6.58	4.27	3.98E-02	6.35E-01
HBB.B1	15	2.97	4.18	4.26	3.99E-02	6.35E-01
GOLGA3	15	4.33	6.10	4.26	4.01E-02	6.35E-01
GATD3	15	4.72	6.67	4.25	4.02E-02	6.35E-01
B2M	15	5.79	8.19	4.24	4.04E-02	6.35E-01
GSS	15	5.76	8.15	4.24	4.05E-02	6.35E-01
PRDX2	15	7.00	9.95	4.22	4.09E-02	6.35E-01
AMBP	15	4.77	6.80	4.21	4.12E-02	6.35E-01
ALB	15	3.39	4.83	4.21	4.12E-02	6.35E-01
PGK1	15	7.00	10.00	4.20	4.14E-02	6.35E-01
KRT2	15	5.87	8.41	4.19	4.16E-02	6.35E-01
RPL3	15	5.11	7.35	4.17	4.21E-02	6.35E-01
RPL18	15	4.46	6.43	4.16	4.24E-02	6.35E-01
HBA1	15	5.32	7.73	4.13	4.33E-02	6.35E-01
PKLR	15	5.74	8.42	4.09	4.43E-02	6.35E-01
PZP	15	0.92	1.36	4.06	4.51E-02	6.35E-01
KLC4	15	7.27	10.78	4.05	4.53E-02	6.35E-01
CENPV	15	4.00	5.94	4.04	4.56E-02	6.35E-01
TTR	15	1.72	2.56	4.02	4.61E-02	6.35E-01
ALDOB	15	7.65	11.43	4.02	4.62E-02	6.35E-01
MIOX	15	6.77	10.12	4.01	4.62E-02	6.35E-01
VCAM1	15	4.46	6.69	4.00	4.67E-02	6.35E-01
HADHB	15	7.38	11.09	3.99	4.68E-02	6.35E-01
FASN	15	7.26	10.93	3.99	4.70E-02	6.35E-01

Supplemental Table 3: Proteome wide correlations with functional outcomes

	tGFR_28day	NGAL_28day	BUN_24hr	BUN_28day	Cr_24hr	Cr_28day	CystatinC
DLAT	0.16	-0.12	0.53	0.06	0.29	0.26	0.36
TTR	0.42	-0.26	0.53	-0.05	0.38	0.41	0.65
ALB	0.11	-0.32	0.58	0.09	0.35	0.43	0.68
FBN1	-0.49	0.10	0.48	0.51	0.76	0.27	0.37
COL12A1	-0.48	0.16	0.49	0.51	0.74	0.34	0.40
KRT19	-0.35	0.10	0.26	0.44	0.60	0.35	0.29
UMOD	-0.34	0.08	0.49	0.45	0.71	0.31	0.47
COL5A1	-0.28	0.20	0.39	0.43	0.73	0.30	0.41
H1.4	-0.49	-0.04	0.57	0.50	0.51	0.36	0.43
PKLR	-0.26	-0.14	0.67	0.36	0.56	0.40	0.52
CP	0.09	-0.08	0.48	0.41	0.47	0.38	0.50
AMBP	-0.37	0.65	-0.29	0.50	0.16	0.32	-0.33
APOA4	-0.36	0.39	-0.14	0.34	0.00	0.48	-0.29
SLC22A8	0.47	0.43	-0.64	0.08	-0.70	-0.25	-0.75
NEDD4L	-0.15	0.25	-0.48	-0.19	-0.52	-0.36	-0.58
MLYCD	0.19	0.03	-0.62	-0.29	-0.65	-0.27	-0.50
CYP4A12A	0.34	0.24	-0.52	-0.30	-0.47	-0.49	-0.49
EPB41	0.68	-0.35	-0.16	-0.50	-0.37	-0.07	-0.07
GK	0.66	-0.22	-0.15	-0.70	-0.68	-0.40	-0.28
DAZAP1	0.66	0.15	-0.12	-0.19	-0.15	-0.06	-0.05
SPTA1	0.65	-0.70	0.32	-0.48	-0.09	0.02	0.50
PLEKHA6	0.64	0.42	-0.36	-0.06	-0.29	0.17	-0.35
NPEPL1	-0.32	-0.19	0.60	-0.19	0.34	-0.24	0.37
PLG	-0.09	0.08	0.55	0.22	0.51	0.46	0.68
IGFBP7	-0.56	0.22	0.51	0.50	0.81	0.44	0.48
CAPG	-0.23	0.23	0.45	0.46	0.79	0.54	0.48
DPYSL3	-0.38	0.27	0.34	0.56	0.75	0.45	0.43
TPM2	-0.20	-0.03	0.12	0.53	0.43	0.71	0.17
GIGYF2	0.14	0.14	0.08	0.13	0.21	0.70	0.28
MRPS22	-0.17	0.09	0.19	0.02	0.34	0.70	0.32
CTSZ	-0.07	0.15	0.33	0.36	0.65	0.69	0.41
LTBP4	-0.27	0.24	0.13	0.73	0.64	0.42	0.15
NQO1	-0.25	0.33	-0.03	0.71	0.41	0.54	0.00
FBLN7	-0.50	0.31	0.07	0.66	0.50	0.24	-0.08
H1.1	-0.58	0.16	0.27	0.65	0.57	0.24	0.19
CORO1A	-0.36	0.22	0.37	0.62	0.76	0.50	0.43

NUP43	0.15	0.64	-0.26	0.45	0.13	0.26	-0.21
NDUFA5	-0.37	0.64	-0.12	0.34	0.09	0.18	-0.19
RPL10	-0.09	0.64	0.01	0.00	0.08	0.05	0.01
UQCRH	-0.02	0.59	-0.16	0.10	-0.06	0.18	-0.11
ANK1	0.64	-0.57	0.17	-0.43	-0.24	-0.15	0.39
COL4A2	-0.04	-0.10	0.54	0.07	0.38	0.02	0.47
TNC	-0.56	0.22	0.36	0.57	0.75	0.34	0.31
RNH1	0.05	0.12	0.16	0.16	0.42	0.69	0.25
BAIAP2	0.11	0.56	-0.39	0.23	-0.28	0.02	-0.36
DAB2	0.63	0.03	-0.30	-0.22	-0.34	0.19	-0.29
F2	-0.07	-0.16	0.54	0.31	0.53	0.76	0.73
KRT20	-0.57	0.11	0.38	0.60	0.75	0.37	0.41
DCPS	0.15	-0.14	0.39	0.27	0.32	0.66	0.43
CAPNS1	-0.13	0.43	0.27	0.60	0.61	0.15	0.20
ACOT8	-0.12	0.56	-0.44	0.25	-0.20	0.11	-0.58
